# Immune Ecosystem of Virus-Infected Host Tissues

**DOI:** 10.3390/ijms19051379

**Published:** 2018-05-06

**Authors:** Mohamed Maarouf, Kul Raj Rai, Mohsan Ullah Goraya, Ji-Long Chen

**Affiliations:** 1 CAS Key Laboratory of Pathogenic Microbiology and Immunology, Institute of Microbiology, Chinese Academy of Sciences (CAS), Beijing 100101, China; mohamed_maarof@im.ac.cn (M.M.); kulrajrai701@gmail.com (K.R.R.); 2 Key Laboratory of Fujian-Taiwan Animal Pathogen Biology, College of Animal Sciences, Fujian Agriculture and Forestry University, Fuzhou 350002, China; goraya_uaf@yahoo.com

**Keywords:** virus, immunity, ecosystem, virus-host interaction, virus pathogenesis, immune subversion

## Abstract

Virus infected host cells serve as a central immune ecological niche during viral infection and replication and stimulate the host immune response via molecular signaling. The viral infection and multiplication process involves complex intracellular molecular interactions between viral components and the host factors. Various types of host cells are also involved to modulate immune factors in delicate and dynamic equilibrium to maintain a balanced immune ecosystem in an infected host tissue. Antiviral host arsenals are equipped to combat or eliminate viral invasion. However, viruses have evolved with strategies to counter against antiviral immunity or hijack cellular machinery to survive inside host tissue for their multiplication. However, host immune systems have also evolved to neutralize the infection; which, in turn, either clears the virus from the infected host or causes immune-mediated host tissue injury. A complex relationship between viral pathogenesis and host antiviral defense could define the immune ecosystem of virus-infected host tissues. Understanding of the molecular mechanism underlying this ecosystem would uncover strategies to modulate host immune function for antiviral therapeutics. This review presents past and present updates of immune-ecological components of virus infected host tissue and explains how viruses subvert the host immune surveillances.

## 1. Introduction

The immune ecosystem of a virus-infected host can be defined as the systemic interaction between the virus and the host immune system, resulting in either viral clearance or immune-mediated host tissue injury [1,2]. Remarkable havoc to people’s health can stem from highly mutative viruses. Viruses are ever evolving to subvert the immune response, causing emerging, adventitious, or even catastrophic diseases [3].

The immune system is continuously under viral assault, and to counter the invasion threat, it is well known that mammalians have developed a strong innate immune system and an intricate and specialized adaptive immune system to counteract the highly evolving virus infections. Immune system evolution occurs in all hosts from unicellular to vertebrates, for example, viral infection in protozoa leads to viral replication without apoptosis, while in multicellular organisms, apoptosis occurs during viral infection and vertebrate’s response is more complex leading to long term immunity against the invading virus [4,5,6]. With the continuous efforts to uncover complex immune ecosystem mechanisms, scientists have innovated new tools to strengthen the immune system and neutralize the viral infections. For example, introduction of vaccines utilizes the ability of immune systems to produce powerful effective antibodies that can selectively neutralize immunogenic agents. Thus, vaccines enhance the host adaptive immunity and make the host able to counteract invasions of viruses to which it has never been naturally exposed before [1]. Better understanding of how the innate immune system works and how it evolves to fight the continuously mutating pathogens started with the pattern recognition hypothesis proposed in 1989 [7]. Briefly, pathogens carry molecular signatures called pathogen associate molecular patterns (PAMP), which can be recognized by pattern recognition receptors (PRRs) of the host cells. Upon interaction with PAMPs, PRRs become activated and trigger a cascade of immune responses against the viral infection. To date, a number of PPRs have been identified. For example, the toll-like receptor (TLR) family can recognize a variety of different PAMPs [8]. Single-stranded RNA (ssRNA) can function as ligands for TLR7/8, thus sensing RNA virus infections. On the other hand, double-stranded RNA (dsRNA) serve as a ligand for TLR3, thus sensing the viral replication [9,10,11,12]. In addition, host cells are able to detect viral infection through TLR-independent signaling pathways involving other cytoplasmic RNA helicase proteins, such as retinoic acid inducible gene (RIG-I) and melanoma differentiation-associated gene 5 (MDA5), etc. [13]. Immune sensing through innate signaling of virus-infected cells eventually induces the translation of interferon (IFN), these secreted IFNs bind with their cognate receptors present on the cell membrane. This binding activates a signaling pathway leading to expression of various IFN-dependent antiviral molecules [14]. Furthermore, adaptive immunity carries on the duty of viral clearance in the latter stages.

In spite of the existence of several correlative host defense lines to neutralize invasive pathogens or restrict the viral life cycle, viruses have evolved and adapted diverse counter strategies to evade the host immunity, sustaining continuous replication and endurable infection in the host using avoidance and escape tactics. Some viruses have developed highly complicated mechanisms such as viral growth in immunologically privileged sites, e.g., herpes virus establishes a latency in sensory neurons to avoid immune surveillance [15]; antigenic drift, e.g., influenza virus uses this to evade the B-cell immunity [16,17]; induced expression of some factors against innate immunity, e.g., human immune deficiency virus (HIV) can block the interferon induction in dendritic cell using its proteins Vpr and Vif [18,19,20,21,22,23,24]; and other more diverse and intricate maneuvers.

In this review, we make a comprehensive overview of virus-host interactions, especially immune ecosystem of virus-infected host. We also highlight the virus subversion mechanisms against host immune responses.

## 2. Immune Sensing of Viral Infection

Host immune response is first activated by sensing the viral infection through PRRs [25]. The host cells interact with the virus in several different viral states, including extracellular native virus, intracellular viral components, and the viral replicate intermediate. Immune sensing of viral infection is mainly achieved by specialized cells and cellular factors to detect specific viral elements in different viral forms [26].

The immune response differs according to type of virus and route of infection [27]. For instance, a study showed that upon infection with inactivated whole influenza virus vaccine containing viral ssRNA, the TLR7/myeloid differentiation primary response 88 (MYD88) pathway was the only pathway triggered without any activation of the RIG-I/IPS-1 pathway, though both of them are parallel innate immune pathways [28]. For DNA viruses, TLR9 can recognize the unmethylated DNA and share the downstream signaling pathway with the adapter protein MYD88 [29]. Viral DNA is also recognized by cGAS in the cytoplasm and IFI16 in the nucleus. Both cGAS and IFI16 recruit a common adaptor protein, Stimulator of interferon genes (STING), which signals to TBK1 for the activation of IRF3, which ultimately induces the expression of type I IFN and other antiviral genes [30]. However, the innate immune response against a DNA vaccine containing CpG-DNA was triggered only by the TBK1 not the TLR9/MYD88 pathway [31].

## 3. Intercellular Immune Ecosystem of Virus-Infected Tissues

In multicellular organisms, cells are actively working as a single fundamental unit countering the viral infection. Once the virus attaches to host cells, the host cells starts a series of events to alert the neighboring cells against the invader and trigger the effector cells and pro-inflammatory response. This happens via the production of cytokines which help the neighbor cells to produce some inhibitory effects on viral infection and replication, e.g., IFNs; a potent neutrophil chemoattractant, e.g., CXCL8; other cell chemoattractants (monocytes, eosinophils, and T cells) e.g., CCL2, CCL3, CCL4, and CCL5; or other cytokines that lead to the acute-phase viral removal, e.g., interleukin (IL)-6 [32,33]. Knowing that the pathogen replication speed is a cornerstone in the viral pathogenesis, establishment of such an intercellular immune ecosystem including intercellular interaction and intracellular signaling is considered beneficial to host antiviral defense, which is able to clear the pathogen and limits its spread in infected tissues without waiting for the classical immune response [34].

Not only viruses are undergoing modifications and development, the tissue’s microenvironment also undergoes several modifications after a successful infection resolution. These modifications can be in favor of the next infection or to counter it. For example, after severe lung infection, severe lung tissue damage leads to a repair process that changes the lung matrix composition, (such as more collagen and fibronectin deposition) providing additional binding sites for bacteria [35]. Successive lung infections can change the lymphatic network and the frequency of inducible bronchus-associated lymphoid tissue (iBALT) [36,37]. In addition, specific memory T cells persist at the infection site and are termed resident memory T cells. These cells are resident within the infection tissues and can promote the early innate immune activation for the recurrent infection. Skin resident CD49a^+^ cells were reported to express perforin and granzyme B after treatment with IL-15 [38,39,40].

It is worth mentioning that natural killer (NK) cells are critical for early non-specific resistance against the viral infection [41]. NK cells are specialized lymphocytes lacking antigen-specific receptors, yet they are able to demolish tumor cells, virus-infected cells, and any cell in the state of stress [42]. NK cells are able to differentiate between normal healthy cells and abnormal cells via certain sophisticated attributes of the cellular surface receptors. The leading receptor for NK cells is the class I major histocompatibility complex (MHC); unstable expression of the class I MHC means an unbalanced cellular state resulting via serial of cascades in the activation of NK cells [43]. However, receptors such as NKG2D and NKp30 are known to help the NK cells to differentiate between healthy and unhealthy cells [44,45]. NK cells have a higher tendency to lyse cells lacking surface class I MHC expression. NK cells lyse the virus-infected cells with the help of cytotoxic T lymphocytes (CTL), inducing cellular apoptosis using its cytolytic granules (containing perforin, granzyme A, and granzyme B) [46].

In the host-virus ecosystem, the virus has evolved counter defenses to help in the continuity of the infection cycle. Some viruses have developed strategies to delude the NK cells or disrupt the class I MHC antigen presentation, thus curbing the NK cells. For example, poliovirus protein 3A interacts with the endoplasmic reticulum (ER) membrane to cease protein transport from the ER to the Golgi apparatus, hence preventing the transport of the MHC-bearing polio-specific peptide to the cell membrane [47]. Foot and mouth disease (FMD) inhibits protein transport using its viral 2BC protein [48]. The Tat protein of retroviruses interferes with class I MHC messenger RNA (mRNA) transcription [49]. Relocalization of the class I MHC to the *trans*-Golgi network by the retrovirus Nef protein results in the downregulation of the surface expression of MHC-I [50]. Cytomegaloviruses (CMV) can resist the NK cells attack though the severe down-regulation of the class I MHC expression by down-regulation of important proteins (e.g., UL18) that are required for the NK cell stimulation [51]. Primarily, the HLA-C and HLA-E molecules protect normal cells from the NK mediated cell lysis. It was believed that retroviruses like HIV-1 can selectively disrupt the expression of HLA-A and HLA-B but not HLA-C and HLA-E, thus, the infected cells are less likely to be killed by NK cells [52]. Yet, recent research found that HLA-C is downregulated by most primary HIV-1 clones. The viral Vpu protein was reported in this study to reduce the ability of HLA-C restricted CTLs to suppress viral replication in CD4^+^ cells in vitro [53]. Herpesviruses possess a special strategy to coexist with the immune system by encoding several genes that interfere with the MHC-I antigen presentation [54,55,56]. Alphaherpesviruses exploit the fact that neurons are immunologically privileged and have a lower expression of class I MHC compared to other cells; they start a long life latent infection in neurons and express no protein during their latency, thus escaping the CTL immune surveillance [57]. Epstein-Barr virus does express a latency protein but with a glycine-alanine repeats domains that bind to proteasomes, making the latency protein undetectable by class I MHC [58].

It was believed that only the B and T lymphocytes of the adaptive immune response can possess memory that is able to recognize a repeated infection, until several studies challenged this hypothesis [59]. Studies introduced the new term “trained immunity” that describes the enhanced immune response following previous exposure to some immunogenic agents leading to a robust response against related or unrelated pathogens in the recurrent infection [60,61]. Some studies suggested that NK cells could keep a memory of the previous antigens to make them able to mediate a more robust immune response [62,63,64]. Also, monocytes have been reported to possess the same phenomena [65].

## 4. Intracellular Immune Ecosystem of Virus-Infected Cells

**Figure 1:**
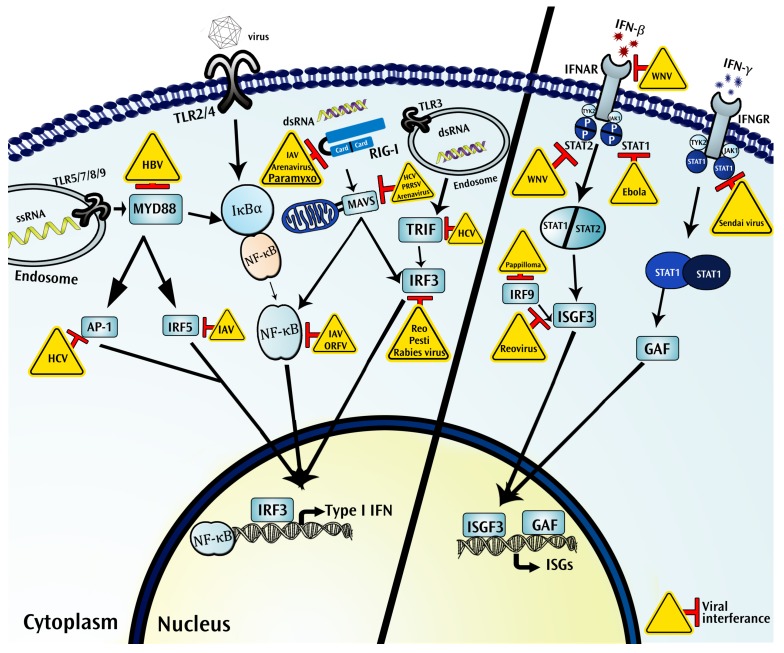
Intracellular immune ecosystem in a virus infected cell. Shown are systemic interactions between virus and host intracellular components involved in the general signaling pathways to induce interferons (IFNs) and IFN-inducible antiviral genes (ISGs). Briefly, upon detection of pathogen associate molecular patterns (PAMPs) by pattern recognition receptors (PRRs), PRR-dependent signaling activates intracellular transcriptional factors such as nuclear factor κB (NF-κB), interferon regulatory factors (IRFs), and others. Activated transcriptional factors translocate into the nucleus and induce type I IFN production. IFN binds to their respective receptors (the interferon-α/β receptor (IFNAR)/IFN-γ-R) to induce activation of the janus kinase-signal transducer and activator of transcription (JAK-STAT) pathway, which in turn activates the transcription complex. Activated transcriptional factors (interferon-stimulated gene factor 3 (ISGF3)/gamma interferon activation factor (GAF)) mediate in the induction of antiviral ISGs. However, viruses have evolved various strategies to counter against antiviral immunity or hijack cellular machinery to survive inside a host cell for their multiplication. Interacting viruses are shown in the yellow triangles. (Ap-1: Activator protein 1; GAF: Gamma interferon activation factor; HBV: Hepatitis B virus; HCV: Hepatitis C virus; IAV: Influenza A virus; IFNAR: The interferon-α/β receptor; IFNGR: Interferon-gamma receptor; IRF: Interferon regulatory factors; ISGF3: Interferon-stimulated gene factor 3; ISGs: interferon-stimulated genes; IκBα: nuclear factor of kappa light polypeptide gene enhancer in B-cells inhibitor, alpha; JAK: Janus kinase; MAVS: Mitochondrial antiviral-signaling protein; NF-κB: nuclear factor kappa-light-chain-enhancer of activated B cells; ORFV: Orf Virus; PRRSV: Porcine reproductive and respiratory syndrome virus; Pesti: Pestivirus; RIG-I; retinoic acid-inducible gene I; Reo: Reovirus; STAT: Signal transducer and activator of transcription; TLR: Toll-like receptors; TRIF: TIR-domain-containing adapter-inducing interferon-β; TYK: Tyrosine kinase; WNV: West Nile virus).

As viruses are obligate intracellular parasites, infected host tissue serves as a central immune ecological niche during viral genome transcription, replication, and stimulation of the host immune response via molecular signaling. The crucial step in the process of the viral invasion is the attachment, which eventually leads to viral recognition by the immune system. Several endocytic pathways are involved in the interaction with the infectious viral components. As a result, the endosomal sensors are an important spot for innate immunity (Figure 1). The immune response can be activated either by detection of the viral PAMPs or the immune and inflammatory cytokines.

Based on the nature of nucleic acid, viruses are classified into DNA and RNA viruses. The majority of viruses infecting animals are RNA viruses. All RNA virus replication proceeds through a RNA strand complimentary intermediate, except for in retroviruses where the intermediate is DNA [66]. Despite differences in genomic features and replication strategies, immediately after virus infection, all RNA viruses trigger evolutionarily conserved innate immune responses that serve as a first line of defense against infection. Immune receptor PRRs, comprised of the key families TLRs, RIG-I like receptors, nucleotide-binding oligomerization (NOD) like receptor, and others, recognize specific PAMPs and thereby stimulate multiple signaling molecular cascades and induce transcription of nonspecific immune effector genes [67]. It is important to know that sensing of the DNA viruses needs a different set of receptors and immune signaling pathways that have already been reviewed in detail (see review in [68]). The difference between the immune sensing and response between the RNA and DNA viruses was also reported and reviewed in detail (see review in [68,69]). Additionally, non-coding RNAs (ncRNAs) have recently gained wide research interest. In addition, the key roles of ncRNAs in the immune response against viral infections has been established and reviewed already (see review in [12,13,70,71,72]).

Importantly, sensing of PAMPs by PPRs remarkably up-regulates the genes involved in the inflammatory response encoding pro-inflammatory cytokines/chemokines and interferons (IFNs) that induce antiviral gene products (Figure 1) [73]. Production of type I IFNs plays an important role in the induction of antiviral responses, which triggers transcription of IFN-inducible antiviral genes (ISGs) (Figure 1). On the other hand, viruses have evolved several strategies to hijack the host cellular machinery and, then, shut off the host cell gene expression at both the transcriptional and translational level. For example, the influenza A virus (IAV) has been reported to induce the degradation of eukaryotic translation initiation factor 4B [74]. Some viruses encode proteins to protect viral nucleic acid from being detected by cytoplasmic sensors. PRR cGAS can sense the HIV viral complementary DNA (cDNA) in the cytoplasm; HIV-1 but not HIV-2 cDNA is protected within the viral capsid until it is translocated to the nucleus for replication. That is due to the affinity of the HIV-1 capsid for stabilization by the host protein cycophilin A (CypA), preventing its exposure to the cGAS in the cytoplasm [75]. Moreover, viruses use a variety of strategies to subvert the interferon response, and these are discussed later in this review [76].

### 4.1. Pattern Recognition Receptors

#### 4.1.1. Toll Like Receptors

TLRs are innate immune recognition receptors acting as the primary sensors of various pathogens [69]. RNA viruses are recognized by TLR 2/3/4/7/8 [10,11,77,78] while DNA viruses are recognized mainly via TLR9 [79,80]. All TLRs activate major cytosolic signaling pathways, mitogen-activated protein kinases (MAPKs), one or more interferon regulatory factors (IRFs), and nuclear factor κB (NF-κB). For instance, intracellular TLRs such as TLR3, TLR7, TLR8, and TLR9 sense pathogen-derived nucleic acids produced inside the host cell upon endocytosis or autophagy. Except TLR3, TLRs are MYD88 dependent for signaling. MYD88-dependent TLR signaling activates transcription factor activation protein1 (AP1), NF-κB, interferon regulatory factor 1 (IRF1) and IRF5. This results in the expression of subsequent pro-inflammatory cytokines [81]. TLR3 signaling is unique, i.e., TIR-domain-containing adapter-inducing interferon-β (TRIF)-dependent. Upon sensing virus-derived dsRNA, TLR3 activates the TRIF-dependent pathway to induce type I IFNs and cytokines [81]. This signaling activates IRF3. Cell type specific network of MYD88 signaling following TLRs activation may lead to the expression of IFN7-dependent large amounts of type I IFNs [82].

Although TLRs are the primary host defense sensors to combat viral invasion, viruses have evolved to subvert TLR-medicated antiviral immunity. Xagorari and Chlichlia [9] reviewed the perturbation of TLR-medicated immunity by numerous viruses [9]. For example, the P protein of the measles virus suppresses TLR signaling through up-regulation of the ubiquitin modifying enzyme A20 [83]. Hepatitis C virus (HCV) inhibits activation of NF-κB and IRF3 by proteolysis of TRIF (the adaptor protein, which links TLR3 and kinase). HCV utilizes the viral NS3/4A to cleave TRIF, which is an intermediate in TLR3, mitochondrial antiviral-signaling protein (MAVS), and RIG-I signaling pathways, therefore, dsRNA cannot induce the IFN production through these pathways [84,85,86]. Recently, an Orf virus (ORFV) virion-associated protein, ORFV119, was identified that inhibits the NF-κB signaling very early in infection [87].

As described above, TLR are mostly endocytic viral nucleic acid sensors [88]. Yet, TLR2 and TLR4 are classical microbial PRRs that recognize microbial moieties and are examples of the surface receptors involved in the recognition of the viral proteins and trigger the pro-inflammatory responses [89]. For example, TLR4 was reported to be triggered by infection with retrovirus, respiratory syncytial virus, and mouse mammary tumor virus [78,90]. TLR2 can detect the hepatitis C virus (HCV), herpes simplex virus (HSV), human cytomegalovirus, and measles particles [91,92,93]. PRRs-dependent activation of signaling pathways is critical for the intracellular immune ecosystem of virus-infected cells.

#### 4.1.2. RIG-I Like Receptors

RIG-I like receptors (RLRs) are cytosolic intracellular key receptors, classically responsible for sensing non-self RNA signatures. Yet, several studies suggested that RIG-I is required to sense the DNA viruses for the induction of type I IFN [94,95,96]. The RLR family, comprised of RIG-I, MDA5, laboratory of genetics and physiology 2, and a homolog of mouse D11lgp2 (Laboratory of Genetics and Physiology 2 (LGP2)), expressed in most of the tissue types (see review in [84]). RLRs contain a central DExD/H-box helicase domain and a C-terminal domain responsible for binding viral RNA. RIG-I and MDA5 share structural similarities. They have two N-terminal caspase activation and recruitment domains (CARDs) for downstream signaling. LGP2 lacks the CARDs and is thought to play a regulatory role in RLR signaling [97].

RIG-I plays an important role in the detection of orthomyxoviruses, rhabdoviruses, and arenaviruses, and MDA5 preferentially detects picornaviruses. Additionally, many other viruses such as flaviviruses, paramyxoviruses, reoviruses, and others are sensed by both RIG-I and MDA5 [98]. Upon detecting the viral RNA ligands in the cytoplasm, RLRs trigger innate immunity and inflammation. The role of RLRs mediated immune signaling in influenza virus infection has been already reviewed with highlights of the important downstream key immune components involved in the activation of IRFs and NF-κ-B [13,99]. An important feature of RIG-I and MDA5 mediated non-self RNA sensing is the activation of the transcriptional factors such as IRF3/7, NF-κB, and Activating transcription factor-2 (ATF2)/c-Jun to induce the transcription of IFNs and pro-inflammatory cytokines. Activation of these transcriptional factors eventually induces the expression of hundreds of ISGs [99].

Viruses show a variety of strategies to evade RLR-mediated immune responses. Importantly, the NS1 protein of the influenza A virus inhibits the functional RIG-I and RIG-I dependent activation of NF-κB [100]. Z proteins of pathogenic arenaviruses showed the ability to interact with RIG-I and MDA5, causing the inhibition of the latter two and leading to a significant inhibition of type I interferon (IFN) responses [101]. The non virion (NV) protein of fish Novirhabdovirus showed the ability to counteract RIG-I and TBK1-dependent interferon and IFN-stimulated gene promoter induction in fish cells resulting in the suppression of the anti-viral state induction [102]. V proteins of paramyxoviruses interact or inhibit MDA5 and LGP2 [103,104]. HCV protease NS3/4A cleaves MAVS at Cys-508 resulting in the dislocation of the N-terminal fragment of MAVS [105]. Some positive-sense RNA viruses like porcine reproductive and respiratory syndrome virus (PRRSV) can cleave the MAVS during infection using their nsp4 cysteine protease [106,107]. Recently, the MDA5-Mediated innate immune response has been reported to be disrupted by different proteins encoded by some picornaviridae viruses, resulting in the inhibition of the viruses interaction with MAVS, which was followed by inhibition of the MDA5-dependent translation of type I IFN [108].

#### 4.1.3. NOD-Like Receptors (NLRs)

NLRs are cytosolic PRRs. They detect diverse PAMPs or damage associated molecular patters (DAMPs) produced by viral infection. The human genome encodes 22 NLRs. Of them, nucleotide-binding oligomerization 2 (NOD2) and multi-protein inflammasome complex (i.e., NOD-, LRR-and pyrine domain-containing 3 (NLRP3)) are well studied for sensing the viral infections. NOD2 was reported to induce IFNs production following ssRNA transfection and respiratory syncytial and influenza A virus infection, whereas NLRP3 inflammasome activation was reported in viral infections such as IAV, encephalomyocarditis virus, and HCV [109,110]. Regulation and activation NLRP3 inflammasomes by viroporins of animal viruses have been already reviewed (see review in [111]). NLRP3 inflammasomes are activated by damage, inflammation, or stress, and this activation leads to the production of active IL-1β and IL-18 by activating caspase I in IAV infection, while NLRP3 can activate the production of cytokines by triggering signal 1 and signal 2 during infection with IAV [13]. The V protein of measles and the NS1 protein in influenza A virus are representative examples of inhibition of the NLRP3 inflammasome [112,113].

### 4.2. Interferons Function as Critical Components in the Immune Ecosystem of a Virus Infected Tissue

Interferons (type I to III) are critical immune-ecological components produced by virus-infected cells; they function as antiviral molecules and immune-modulators. Roles of type I IFNs (IFN-α and IFN-β) are well characterized and intensively explored. IFNs bind to their respective receptors to activate the Janus kinase-signal transducer and activator of transcription (JAK-STAT) pathway that governs antiviral defense [114]. IFNs-activated immunity determines the extent of host susceptibility towards viral infection. Recently, mice lacking interferon α receptor (IFNAR) (type I IFN receptor) were reported to be highly susceptible to pseudorabies virus infection [115]. Remarkably, binding of type I and type III IFNs to their respective receptors activates tyrosine kinase 2 (TYK2) and Janus kinase 1 (JAK1). This activation leads to the recruitment and phosphorylation of signal transducers and activators of transcription, signal transducer and activator of transcription 1 (STAT1) and STAT2. Phosphorylated STAT 1 and STAT 2 hetero-dimerize and are assembled with IFN-regulatory factor 9 (IRF9) to form a tri-molecular complex called IFN-stimulated gene factor 3 (ISGF3). The complex, then, translocates into the nucleus. Inside the nucleus, ISGF3 binds to its cognate DNA sequences, which are known as IFN-stimulated response elements (ISREs; consensus sequence TTTCNNTTTC), thereby, directly activating the transcription of ISGs (see review in [116,117]). On other hand, type II IFN binds to its receptor (IFN-γ receptors 1 and 2 heterodimers) and leads to the formation of phosphorylated STAT1 (pSTAT1) homo-dimers. Phosphorylated STAT1 homo-dimers form the IFN-γ activation factor (GAF). Following GAF nuclear translocation, GAF binds to the gamma-activated sequence (GAS, TTCNNNGAA) in the promoter region of the ISGs, and this results in the expression of antiviral ISGs [116,117]. Furthermore, IFN-γ plays a pivotal role in regulating the immune function and bridging the innate and adaptive immune responses [118]. Importantly, non-canonical/alternative pathways have been emerging to uncover hidden components of the immune-ecosystem. For example, an alternative STAT signaling pathway acts in antiviral immunity in Caenorhabditis elegans [119].

Activated JAK1 also activates other members of the STAT family (STAT1-6) [120] and induces several alternative signaling pathways [13,76,104,109,121]. Activation of all pathways, eventually, further amplifies the amplitude of IFN production and signaling. Networks of cellular pathways that regard IFNs as critical immune components in virus infection have been extensively reviewed elsewhere (see review in [72,122]). IFN-mediated pathways obviously stimulate expression of hundreds of ISGs by activating the specific transcriptional regulator factors. Representative ISGs are MxA, protein kinase R (PKR), 2′-5′ Oligoadenylate Synthetase (OAS), ISG15, viperin, tetherin, IFTIMs, RIPK2, IFI16, and so on [14,32,70,123,124,125]. Recently, many more functional ISGs have been identified through protein-wise or genome-wise screening. Nevertheless, arguably, intensive studies are required to define the roles of these ISGs in the immune ecosystem of a virus infected host.

Although IFNs act as critical immune components, still, viruses can perturb the IFN-mediated immune barriers. The balance of immune ecosystems is always unsteady. Supportive and virtual host immunity is always perturbed by certain types of viruses [76]. Recently, tetherin has been shown to inhibit type I IFN via targeting MAVS [18]. A viral-hijacked E3 ubiquitin ligase is also shown to shut off IFN signaling [33,126]. A variety of IFNs subversion strategies by viruses have been extensively reported over the past 10 years [76]. Importantly, numerous viruses exploit suppressors of cytokine signaling (SOCSs) to inhibit IFN signaling [34,127]. The suppression of type III IFN signaling by virus-induced SOCS-1 was reported to cause an adaptive increase in type III IFN expression by the host to protect cells against the viral infection, as a consequence, it lead to excessive production of the IFNs with impaired antiviral response [118]. Some viruses can encode antagonists for type I IFN, such as the vaccinia virus that embraces the C9 ankyrin repeat/F-Box protein that has been recently considered as an antagonist of the Type I IFN-induced antiviral state [128]. Kaposi’s sarcoma-associated herpesvirus (KSHV) can selectively interact with IRF7, inhibiting the IRF7 dimerization and leading to the suppression of IRF7-mediated activation of type I IFN [129]. The pestiviral protein N (Npro) was reported to interact with the IFN regulatory factor 3 (IRF3) via binding to the active form of IRF3 in the presence of its transcriptional coactivator, CREB-binding protein (CBP), resulting in the inhibition of the activation of type I alpha/beta IFN [130]. DNA viruses such as adenovirus, human papilloma virus, and Kaposi’s sarcoma virus utilize their viral proteins to bind with the DNA adaptor STING to prevent induction of type I IFN [131,132]. Viruses have also evolved to inhibit the action of ISGs. For example, protein kinase R (PKR) is one of the antiviral effector ISGs; human cytomegalovirus virus encodes pTRS1 and pIRS1 proteins that antagonize PKR, preventing its autophosphorylation of eukaryotic initiation factor 2 alpha (eIF2α) and facilitating the viral replication [133]. Vault RNAs (vtRNAs) have been reported to promote IAV replication via the inhibition of PKR activation and the subsequent IFN response [125]. Taken all together, virus-induced IFNs production and suppression of their signaling seem to be in the state of delicate and dynamic equilibrium by modulating host factors to maintain a balanced immune ecosystem [12]. Destruction of such a balanced immune ecosystem is a primary cause of viral pathogenesis.

## 5. Adaptive Immune Response to Viral Infection

The primary adaptive immune response usually takes several days and starts with the binding of an antigen with its specific receptor on the T cells or B cells. Migration of the pathogen stimulates dendritic cells of the draining lymph nodes. This is followed by several steps that ends with the release of lymphocytes embracing the antigen-specific receptors and the production of effector and memory cells. The adaptive immune response to viral infection has two-main arms: humoral immunity and cellular immunity. Humoral immunity consists of the antibodies secreted by plasma cells that can neutralize the native extracellular viruses. The cellular immunity is driven by α-β T cell receptors expressed by T lymphocytes that recognize the antigen processed peptide bound to MHC molecules on the surface of infected cells [134,135]. After the primary exposure to a certain pathogen, adaptive immune response confers long-term, often lifelong, protection against the exposed pathogen. This is due to the fact that adaptive immune response exhibits memory T cells or B cells to provide anearly response against the recurrent infection. In case of reinfection, memory B cells are responsible for the generation of an accelerated and more robust antibody-mediated immune response [136]. Memory T cells are the T lymphocytes that were previously exposed to the antigen, either via natural or artificial exposure, so at the second encounter, the memory T cells can reproduce quickly to mount a faster and stronger immune response than was seen during the first infection [137]. Memory T cells are sub-classified into two important types: central memory T cells (T_CM_) and effector memory T cells (T_EM_). T_CM_ is capable of the production of interleukin (IL)-2 and reproduces extensively, while T_EM_ is capable for the production of effector cytokines like IFN-γ [137]. This mechanism allowed great progress in the production of vaccines to deleterious viral infections that impact the economy and public health. Exploiting the adaptive immune response and the memory cells produced by the immune system, we are now able to protect humans, animals, and plants from diseases that they have never been exposed to, via exposing them to an artificial compound containing either conventionally inactivated, live-attenuated virus vaccines, recombinant viruses that express protective proteins of heterologous viruses, virus-like particles (VLPs), or DNA vaccines [134,138].

Various viruses have evolved to invade the effector cells to evade the adaptive immunity. For example, HIV can infect the CD4^+^ T_H_ cells, resulting in a serious immune suppression due to cellular lysis [139]. Measles virus infects B cells, CD4^+^, and CD8^+^ memory T cells and monocytes, resulting in immune suppression that lasts for several weeks after the virus invasion [140]. Epstein-Barr virus infects the B cells, causing impairment of the antigen recognition and antibody release [141]. In addition, some viruses can infect the thymus in the early animal life, leading to its identification as a non-immunogenic antigen and, therefore giving immune tolerance as the virus is no longer recognized as a foreign antigen. This results in a long-life infection of the animal. Examples of these viruses are lymphocytic choriomeningitis virus [142] and murine leukemia virus [143]. On the other hand, hepatitis B virus (HBV) infection has a unique infection style in infants or young children via causing an asymptomatic disease phase (immune tolerant phase) characterized by high HBV titers and a low incidence of liver inflammation and immune response to the virus [144,145]. The mechanisms underlying the immune tolerance is still unclear, however, they might be due to ineffective antigen processing and transport to major histocompatibility complex class I molecules [146] leading to HBV-specific T-cell hyporesponsiveness [147]. HBV can also harness the young children’s developing immune system and make the fetal immunity facilitate its persistence in patients after the prenatal exposure, causing a persistent long-life infection [148]. Many other viruses have developed persistence mechanisms to cause permanent infection and to persist indefinitely within the host [149]. Herpes virus can cause nonproductive infection by developing the herpes virus latency in immune privileged sites [150]; retreoviruses cause their persistent infection via integration of their provirus into the host genome [151]; and some other viruses cause a persist infection through continuous viral replication, e.g., filoviruses [152], arenaviruses [153], and polyomaviruses [154,155].

## 6. Conclusions

Dynamic interaction between the virus and host immune system results in the formation of a complex immune ecosystem involving intermolecular signaling webs for their synergistic or antagonistic fitness and survival. The host cell modulates intracellular components to clear the virus away, but viruses hijack host cell components, simultaneously. To guarantee successful and durable defense mechanisms against virus infection, hosts have evolved a highly intricate, sophisticated, and adaptable immune system to protect against continuously emerging threats and mutated viruses. Antiviral immune responses comprise complex networks of innate and acquired defenders, some of them are specific to certain pathogens and some of them are nonspecific to counteract any state of stress or immunogenic particle. It is well known that innate immunity provides the rapid nonspecific response, while adaptive immunity is only developed after the initial virus exposure. The interplay between the innate and adaptive immunity will provide the desirable immune ecosystem that can challenge the virus infection and demolish the infected cells.

On the other hand, viruses also evolve. Viruses have co-evolved with their hosts to produce remarkable strategies to counteract the host defenses. These strategies include the rapid shutdown of host molecular synthesis, evasive strategies of viral antigen production, interference with MHC class I and class II antigen presentation, impairing NK cell function, suppression of antiviral cytokine signaling, and blocking apoptosis. It is recognized that health can be compromised in are remarkable way by highly mutative viruses. Viruses are ever evolving and being transmitted to susceptible hosts from animal or environmental sources to cause diseases, such as often catastrophic emerging and adventitious diseases. Virus fitness inside a host is critically reliant on the host immune ecosystem and its strategies to counter the immune ecosystem. Approaches in the understanding and management of host immune ecosystem and virus-host community would further develop novel antiviral therapeutics.

## Abbreviations

AP1	Activation Protein1
APC	Antigen Presenting Cells
ATF2	Activating transcription factor-2
CARD	Caspase Activation and Recruitment Domains
CMV	Cytomegaloviruses
CTL	Cytotoxic T Lymphocytes
ds/ssRNA	Double/Single Stranded RNA
ER	Endoplasmic Reticulum
FMD	Foot-and-mouth disease
GAS	Gamma-Activated Sequence
HCV	Hepatitis C Virus
IAV	Influenza A Virus
IFN	Interferon
IFNAR	Interferon α Receptor
IFGAR	Interferon γ Receptor
IL	Interleukins
ISGF	IFN-Stimulated Gene Factor
JAK1	Janus Kinase 1
LCMV	Lymphocytic Choriomeningitis Virus
LGP2	Laboratory of Genetics and Physiology 2
MAPKs	Mitogen-activated Protein
MAVS	Mitochondrial Antiviral Signaling Protein
MDA5	Melanoma Differentiation-associated Gene 5
MHC	Major Histocompatibility Complex
miRNAs	microRNAs
MLV	Murine Leukemia virus
MYD88	Myeloid Differentiation Primary Response 88
ncRNA	Non-coding RNAs
NF-κB	Nuclear Factor κB
NK	Natural Killer
NLRP3	NOD-, LRR- and pyrine domain-containg 3
NOD	Nucleotide-binding Oligomerization
OAS	2′-5′ Oligoadenylate Synthetase
PAMP	Pathogen Associated Molecular Patterns
PI3K	Phospho-Inositol 3 Kinase
PRR	Pattern Recognition Receptors
STING	Stimulator of interferon genes
RIG-I	Retinoic Acid Inducible Gene
RSV	Respiratory Syncytial Virus
STAT	Signal Transducers and Activators of Transcription
TCM	Central memory T cells
TEM	Effector memory T cells
TLR	Toll Like Receptors
TYK2	Tyrosine Kinase 2
WNV	West Nile virus
